# Sickle cell disease implicated in the development of severe dengue: A pediatric case series

**DOI:** 10.1590/0037-8682-0081-2026

**Published:** 2026-04-10

**Authors:** Melissa Reyes Hernández, Ana García Suarez, Hernando Pinzón Redondo, Carlos Moneriz

**Affiliations:** 1 University of Cartagena, Faculty of Medicine, Biochemistry and Diseases Research Group, Cartagena, Colombia.; 2 University of Cartagena, Faculty of Medicine, Department of Pediatrics, Cartagena, Colombia.; 3 Napoleón Franco Pareja Children's Hospital, Pediatric Infectious Diseases, Cartagena, Colombia.

**Keywords:** Sickle cell disease, Dengue virus, Children

## Abstract

Dengue poses a considerable risk to individuals with sickle cell disease. This report presents six cases of male pediatric patients (mean age 9.7 ± 4.8 years) with a hemoglobin S mutation, all of whom exhibited clinical manifestations consistent with dengue infection. Diagnosis was confirmed using NS1 antigen, serology (IgM/IgG), and RT-PCR, identifying all cases as dengue virus serotype 2. All patients had warning signs at presentation; three were severe, four required intensive care, and one died from dengue shock. Early identification and management are critical to reduce mortality in children with hemoglobin S mutations during dengue epidemics.

## INTRODUCTION

Dengue is an acute febrile illness attributed to the dengue virus (DENV), transmitted predominantly by mosquitoes of the *Aedes* genus, particularly *Aedes aegypti*. The World Health Organization (WHO) estimates 100-400 million infections annually, although only approximately one-quarter result in clinically apparent disease^1^. Dengue remains endemic in over 100 tropical and subtropical countries, including Colombia, where cyclical outbreaks reappear every three to four years^2^. Recent data from Colombia’s National Institute of Health indicated a sharp increase in dengue activity during 2024, with cases exceeding 500,000 nationwide^3^. In the Caribbean region-especially Cartagena and Bolívar-incidence and fatality rates have increased, influenced by El Niño-related environmental conditions favorable to *Aedes aegypti* breeding^3^. This significant escalation has heightened concern among local health authorities and highlights the persistent public health challenge posed by dengue in the region^3^.

Dengue presents in various clinical forms. The WHO classifies dengue into three major categories: dengue without warning signs, dengue with warning signs, and severe dengue shock syndrome (DSS)^2^ ^,^ ^4^. Individuals with chronic illnesses such as sickle cell disease (SCD) are at increased risk for serious complications^5^. SCD is a hereditary disorder involving the β-globin gene that produces hemoglobin S (HbS)^4^. This genetic mutation results in decreased erythrocyte flexibility and increased fragility, causing obstruction of small blood vessels. These blockages impair blood flow and oxygen delivery, leading to anemia, episodes of severe pain, and progressive injury to multiple organs^4^ ^,^ ^5^.

In Colombia, dengue continues to pose a considerable burden on public health, particularly in coastal regions with a high concentration of Afro-descent populations and elevated incidence of anemia^3^ ^,^ ^6^. The concurrent prevalence of these two conditions poses ongoing challenges for local health services, underscoring the need for enhanced preventive measures and clinical care strategies^6^ ^,^ ^7^.

Despite continuous efforts, the management of patients with both conditions remains complex. A comprehensive review of the Cochrane Library, LILACS, SciELO, MEDLINE, PubMed, and PMC identified approximately 20 publications related to dengue and SCD; however, only seven studies provided sufficient clinical data for inclusion. Most were case reports and small case series, with four addressing pediatric or adolescent patients. 

This study details the clinical and pathophysiological features of children with HbS mutations treated for dengue at a hospital in Cartagena, Colombia. By providing original evidence on this understudied high-risk phenotype in Latin America, we aim to delineate clinical and laboratory findings that may help identify factors associated with more severe disease. The findings underscore the importance of early identification and differentiated management for children with HbS who contract dengue.

## CASE REPORT

Six male children (mean age 9.7 ± 4.8 years) with confirmed diagnoses of SCD and dengue were reported by the Napoleón Franco Pareja Children’s Hospital (HINFP) in Cartagena, Bolívar Department, Colombia. The diagnosis of dengue was confirmed through positive serological and virological assessments, including detection of NS1 antigen, IgM, and IgG antibodies, and RT-PCR, in accordance with current national guidelines. Sickle cell anemia was previously confirmed by hematological evaluation and hemoglobin electrophoresis as documented in medical records. All were born and raised in Cartagena, belonged to a low socioeconomic stratum, and were insured under a subsidized public health system. All patients presented with dengue with warning signs according to the WHO criteria; three patients (patients 1, 2, and 3) progressed to severe dengue.

Upon arrival at the emergency department, four of the six children exhibited fever (> 38 °C), although caregivers reported that febrile episodes began several days before hospital admission (mean duration: 4.5 ± 1.1 days; Table 1). The most common clinical symptom was abdominal pain (6/6), followed by vomiting (5/6), hepatomegaly (3/6), and jaundice (2/6). Tachycardia was observed in three cases, and tachypnea in two. Less frequent manifestations included retro-ocular pain (2/6), myalgia (2/6), diarrhea (2/6), epistaxis (1/6), splenomegaly (1/6), and rash (1/6). 

**TABLE 1:** Clinical characteristics of six pediatric patients with sickle cell disease and dengue infection.

Clinical manifestation	P1	P2	P3	P4	P5	P6
Dengue classification	Severe	Severe	Severe	With warning signs	With warning signs	With warning signs
Fever (> 38 °C) at admission	+	+	+	+	-	-
Abdominal pain	+	+	+	+	+	+
Vomiting	+	+	+	+	+	-
Hepatomegaly	+	+	+	-	-	-
Jaundice	+	+	-	-	-	-
Tachycardia	+	+	+	-	-	-
Tachypnea	+	+	-	-	-	-
Retro-ocular pain	+	+	-	-	-	-
Myalgia	+	+	-	-	-	-
Diarrhea	-	+	+	-	-	-
Epistaxis	+	-	-	-	-	-
Splenomegaly	+	-	-	-	-	-
Rash	+	-	-	-	-	-
Arthralgia	-	-	-	-	-	-

**P:** patient; **+:** present; **-:** absent.

Laboratory investigations indicated that five patients demonstrated hemoglobin levels below the normal range, consistent with anemia (Table 2). Four presented with leukopenia and thrombocytopenia, and elevated transaminase levels were detected in those with severe dengue. Certain laboratory tests were not performed in some patients due to their stable presentation at admission. All six tested positive for the NS1 antigen, with serological assays confirming the presence of anti-dengue IgM and/or IgG. RT-PCR further verified dengue virus infection, and all patients were identified as having serotype 2 (DENV-2). Hemoglobin variants were detected in all cases. Notably, one patient lacked regular follow-up or treatment for SCD. 

**TABLE 2:** Initial paraclinical and laboratory findings in pediatric patients with sickle cell disease and dengue infection.

Laboratory test	P1	P2	P3	P4	P5	P6
Hemoglobin (g/dL)	9.1	7.9	8	11.3	14.8	11.7
Hematocrit (%)	26.4	23	23.8	34.3	45.9	37.1
Leukocytes (mm^3^)	12300	9800	3200	3500	2700	3000
Neutrophils (%)	78	79	19.8	52.6	50.4	53.4
Lymphocytes (%)	8.4	17.1	73	40.8	44.7	39.8
Platelets (mm^3^)	50,000	73,000	56,000	234,000	158,000	135,000
PT (Sec)	19	11.8	11.8	15.8	NP	NP
PTT (Sec)	30.7	37.7	37.1	39.1	NP	NP
Total bilirubin (mg/dL)	11.2	2.12	1.4	NP	NP	NP
Direct bilirubin (mg/dL)	1.64	0.77	0.65	NP	NP	NP
Indirect bilirubin (mg/dL)	9.56	1.3	0.75	NP	NP	NP
AST (U/L)	10,700	257	829	36	NP	NP
ALT (U/L)	3210	201	473	17	NP	NP
Albumin (g/dL)	3.2	2.8	2.8	3.4	NP	NP
Dengue NS1 antigen	+	+	+	+	+	+
Dengue IgM	+	-	+	+	-	+
Dengue IgG	-	-	+	+	-	+
RT-PCR for dengue virus	+	+	+	+	+	+
DENV serotype	2	2	2	2	2	2
Hemoglobin electrophoresis (%)	HBS: 34	HBS: 82	HBS:37.7	HB S: 49	HBS: 70.6	HBS: 66.4
	HBA1: 64	HBF: 17	HA:59.9	HB A: 43.7	HBA: 25.2	HBA: 28.5
	HBA2: 2	HBA2: 1	HBA2:2.4	HBF: 6.2	HBF: 4.2	HBF: 5.1
Genotype	AS	SS	AS	AS	S/β⁺	S/β⁺

**+:** test positive; **-:** test negative; **NP:** not performed due to clinical stability at presentation; **P:** patient; **Hb:** hemoglobin; **Hct:** hematocrit; **PT:** prothrombin time; **PTT:** partial thromboplastin time; **AST:** aspartate aminotransferase; **ALT:** alanine aminotransferase; **NS1:** nonstructural protein 1 antigen; HbA1, HbA2, HbS, **HbF:** hemoglobin fractions. **AS:** sickle cell trait (heterozygous HbA/HbS); **SS:** sickle cell anemia (homozygous HbS); **S/β⁺:** sickle cell-beta⁺ thalassemia.

The average interval from symptom onset to full recovery was eight days. Four patients required intensive care unit admission and one died despite intervention. Patients 1, 3, and 6 remained at home for approximately 4-5 days after symptom onset, which likely contributed to the detection of dengue IgG at testing. All patients received intravenous fluids; three classifieds as having severe dengue required blood component transfusion, and two received all three components.

All patients and their caregivers were aware of their sickle cell condition. Five children received hydroxyurea, folic acid, and ascorbic acid. Patient 1 was categorized as neglected, as his family did not ensure adequate follow-up or therapy for SCD. The lack of ongoing medical oversight resulted in complications necessitating intensive care unit admission, culminating in multiorgan failure and DSS with subsequent death. The remaining patients recovered and were discharged with instructions to continue follow-up care for their chronic disease.

### Ethical Considerations

This study was approved by the Ethics Committee of the Napoleón Franco Pareja Children's Hospital in Cartagena, Colombia (approval date: May 16, 2025; protocol number: HINFP-2025-160525).

## DISCUSSION

Since initial reports from Cuba, SCD has been recognized as a major risk factor for severe dengue, with mortality rates of up to 30 times higher than in those without SCD^7^. Children, particularly ages 5-9, are also at greater risk of severe outcomes^8^, as noted in fatal cases from Cuba, Jamaica, Brazil, and Colombia^9^, consistent with the outcomes in the current case series. All patients were infected with DENV-2, often linked to severe pediatric outcomes and predominant in Caribbean Colombia^10^.

While most research focuses on children, severe dengue also occurs in adults with SCD. Retrospective analyses indicate increased risks in children and adults with SCD^7^ ^,^ ^9^. However, limited adult-specific data prevent definitive age-based comparisons.

Dengue infections may range from asymptomatic manifestations to febrile illness of varying intensity. Among the patients in this report, four experienced temperatures ≥ 38 °C. Although not all children had a fever at admission, each was carefully monitored due to reported abdominal pain at presentation or during the preceding days. The WHO highlights abdominal pain as a warning sign of severe dengue in pediatric populations^1^, along with symptoms such as vomiting, persistent fever, and hepatomegaly-all of which were documented in several cases within this series.

**FIGURE 1:**
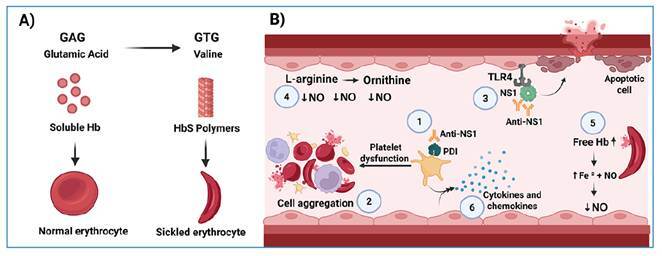
Pathophysiology of dengue infection in patients with sickle cell disease. **A**) Base changes (A ® T) cause a substitution of glutamic acid for valine (GAG → GTG), resulting in hemoglobin S. The polymerization of abnormal globin chains leads erythrocytes to adopt a sickle shape. **B)** (1) Anti-DENV NS1 binds PDI on the platelet surface, altering its activity. (2) Altered sickle erythrocytes, platelets and endothelium overexpress adhesion molecules, carrying erythrocytes, platelets, leukocytes and other plasma components with them, triggering vaso-occlusive crises. (3) Vascular leakage is induced by endothelial glycocalyx degradation and apoptosis of cells that express TLR4, a target of DENV and anti-NS1. (4) Free iron directly binds to and inactivates NO, (5) while arginase released from erythrocytes and endothelial cells converts arginine to ornithine, decreasing NO synthesis and impairing the underlying vasodilatory mechanisms. (6) These conditions in the endothelium, together with their interaction, are crucial for the production of additional cytokines and chemokines, which amplify proinflammatory and procoagulant molecule release. **Hb:** hemoglobin; HbS: sickle hemoglobin; **NS1:** nonstructural protein 1 of dengue virus; **Anti-NS1:** antibody against NS1; **PDI:** protein disulfide isomerase; **TLR4:** toll-like receptor 4; **NO:** nitric oxide; **Fe²⁺:** ferrous iron; **DENV:** dengue virus. *Figure created with BioRender.com.*

In sickle cell anemia, HbS polymerizes as oxygen levels decrease, forming long, rigid filaments within erythrocytes (Figure 1A). This process disrupts key structural proteins-including band 3 protein, ankyrin, and spectrin-thereby altering cell morphology and reducing flexibility^11^. This diminished erythrocyte deformability promotes microvascular occlusion, thereby limiting tissue oxygenation and triggering painful vaso-occlusive crises^11^. Impaired endothelial relaxation mechanisms further exacerbate vaso-occlusion and intravascular thrombosis (Figure 1B)-hallmarks of the disease. The resultant changes in erythrocyte structure also foster cellular aggregation and activation, yielding measurable hematological changes^11^. In this study, three patients had hemoglobin and hematocrit values below reference limits, whereas four had thrombocytopenia. These findings align with expected hematological disturbances in individuals with SCD and dengue infection.

Elevated liver enzymes and altered coagulation profiles in these pediatric cases suggest hepatic injury, increased capillary permeability, and platelet dysfunction. Without timely intervention, these abnormalities can precipitate plasma leakage and acute hypotensive episodes consistent with DSS, as observed in Patient 1. DSS represents the most critical phase of the disease^12^.

Recent research suggests that vascular leakage in dengue is largely mediated by the NS1 protein, which binds to endothelial cells via Toll-like receptor 4 and, upon targeting by anti-NS1 antibodies, contributes to glycocalyx disruption (Figure 1B)^12^. Documented cross-reactivity between these antibodies and antigens on endothelial cells and platelets may trigger apoptosis, potentially explaining the association between disease severity and thrombocytopenia in dengue^12^.

This study has certain limitations, notably its small sample size and single-center retrospective design. Although molecular confirmation and serotyping were performed, larger multicenter prospective cohort studies are warranted to better define the clinical risk factors, immunological mechanisms, and predictors of severe outcomes in this high-risk population. Detecting SCD at birth through neonatal screening and maintaining robust mosquito control and dengue surveillance programs are critical measures to reduce mortality associated with this combination of diseases.
